# Possibilities, usage and needs of an app-based health prevention among seafarers

**DOI:** 10.1371/journal.pone.0310440

**Published:** 2024-11-27

**Authors:** Luciano Arslan, Dorothee Dengler, Lukas Belz, Felix Alexander Neumann, Birgit-Christiane Zyriax, Volker Harth, Marcus Oldenburg

**Affiliations:** 1 Institute for Occupational and Maritime Medicine Hamburg (ZfAM), University Medical Center Hamburg-Eppendorf (UKE), Hamburg, Germany; 2 Preventive Medicine and Nutrition, Midwifery Science ‐ Health Services Research and Prevention, Institute for Health Services in Dermatology and Nursing (IVDP), University Medical Center Hamburg-Eppendorf (UKE), Hamburg, Germany; Vietnam Maritime University, VIET NAM

## Abstract

The present study analyses the technical requirements as well as the user behaviour of seafarers for an app-based health prevention, including apps for wellness, prevention, fitness, medical care and mental well-being. In a maritime field study 976 seafarers on 65 merchant ships participated in the survey carried out with a questionnaire. The vast majority (98.4%) of the respondents had a mobile device on board. 52.5% stated to already have downloaded a health app, with a higher proportion among the officers (adjusted Odds Ratio (aOR) 1.67; 95% CI (1.13–2.50)). The most common reasons for downloading this kind of app were activity tracking (74.8%), weight loss (41.8%) and exercise (41.0%). Officers downloaded apps significantly more often for activity tracking (p< 0.001) and sleep tracking (p = 0.001). 51.1% of downloaders stopped the use of a health app. Frequent reasons for not downloading or stopping the use of a health app were loss/absence of interest and that the desired health apps could not be used offline. Frequency and duration of use were highest at home, followed by the use at sea and then in port. No correlation between the WHO Well-being Index and the use of health apps could be found. Overall, two kinds of obstacles to implementing app-based health intervention could be identified: maritime-specific obstacles and general obstacles (obstacles not exclusively assignable to the naval environment); the maritime-specific obstacles primarily consisted of being offline for long periods of time and limited recreational time. Among the most important general problems were economic and social problems as well as a significant loss/absence of interest. Nevertheless, the basic requirements for an app-based health prevention for seafarers seem to be in place. Measures taken by the shipping company could consist of providing internet access for app usage and educating seafarers on the benefits of health apps. Providing preselected options and guidance on app selection and licenses for paid apps could further encourage usage among seafarers and strengthen success of an app-based health intervention eventually leading to improved physical and mental health in their employees. Additionally, if follow-up maritime studies can objectively demonstrate benefits and positive health effects, the International Maritime Organization (IMO) could officially recommend the use of health apps as a health management measure and play an important political role by recommending and supporting the development of health apps specifically designed for maritime conditions with key features like offline availability.

## Introduction

The profession of seafarers is characterized by numerous physical and psychosocial health hazards, some of which may be associated with occupation-based risks, and others with lifestyle behavior. The most relevant stressors include isolation, separation from family, loneliness on board, physically demanding shift work, reduced sleep quality and duration, limited leisure time activity as well as permanent physical influences such as noise and swell [1–3].

Seafarers often suffer from lack of sleep, fatigue, psychophysical exhaustion [4–6] and are described in the literature as an occupational group with increased risk factors for cardiovascular diseases (CVD) (high blood pressure, elevated blood lipid levels, obesity, e.g.) [7–12]. Smoking is also described as a widespread habit among seafarers [13, 14] and a balanced diet is rarely possible at sea due to job-specific conditions (such as logistics, transport, and costs) which contributes to the already mentioned CVD risk factors [15, 16]. Furthermore, reduced physical activity on board is another lifestyle factor among seafarers associated with increased health risk [17]. For example, seafarers do less exercise on high seas compared to shore leave at home [17].

Due to the demanding work environment and the reduced leisure time opportunities on board, the topic of preventive health care is currently of great importance for seafarers. However, health prevention has only been studied to a limited extent and the evidence base for the effectiveness of health promotion measures on board tends to be scarce [18]. So far, the focus of maritime health intervention has often been limited to a specific target group or health risk, rarely to topics that could lead to a global change in the behavioral patterns of the participants and certain problems of seafarers have been neglected completely [18, 19]. A systematic review supports this assumption [20]: Ten intervention studies were examined that focused on lifestyle changes and health outcomes through structural and educational interventions in a maritime setting. The authors of this review concluded that most of these intervention studies had limited methodological quality and were weakly designed and implemented [20]. The authors of the latter two studies [18, 20] attributed this to the limited possibilities for health promotion on board due to the maritime-specific environment which makes a successful and long-lasting intervention difficult.

The implementation of *digital health prevention* could pose a solution to those maritime-specific obstacles. Measures could be carried out continuously on board and would not depend on the presence of health professionals on the ships or other external factors. The results of a study by Oldenburg & Jensen [21] showed that 89.5% of the examined seafarers owned an internet-enabled cellular phone or computer on board. *Health apps* might pose a good starting point for self-administered and self-responsible health prevention that offers individual, specific solutions since health apps cover a broad area of health prevention topics. These often address the risks of lifestyle-associated causes (such as physical inactivity or tobacco use) as well as the risks arising from the occupational environment (such as stress) and could therefore achieve long lasting behavioral changes in seafarers.

A further systematic review summarized 20 scientific surveys that addressed the issue of health app effectiveness [22]. 16 of the 20 studies showed that apps have had a positive impact on users’ health behavior or health. In addition, there seems to be a general interest in app-based health prevention among seafarers [23] and a Norwegian study found out that for female seafarers apps posed a popular choice for learning about health [24]. Another study investigated the effectiveness of mobile phone-based prevention on skin cancer in Iranian seafarers [25]. The results of this study suggest that a mobile phone-based intervention using risk communication can have a positive impact on preventive behavior.

The findings of an own study investigating the mobile device proficiency of seafarers suggest that the examined seafarers had a mobile proficiency higher than a land-based reference sample in Spain [26]. This finding poses an important basic element to implementing a health app-based intervention, which could have otherwise become a significant obstacle, as it shows that the seafarers´ mobile device proficiency is sufficient for health app usage.

For further investigation of the potential of app-based health promotion in the maritime context, the present study aims to analyze the current possibilities, usage and needs of seafarers towards app-based health intervention.

## Methods

### Design

The data collection was carried out with the help of an electronic self-administered questionnaire that was sent to ship crews of 65 merchant vessels of a German shipping company between the 16^th^ of June and 7^th^ of July in 2021.

### Questionnaire

The questionnaire summarized demographic data, the technical requirements for app-based health management, and the seafarers’ previous usage of health apps. The demographic and lifestyle data were collected with 9 closed questions (*rank*, *age*, *seafaring experience*, *gender*, *family status*, *child status*, *smoking status*, *cigarettes/day*, *alcohol consumption*), and one open question (*nationality*).

Previous health app usage was determined by the already established questionnaire "*characteristics of health app use*" of the study by Krebs & Duncan (2015) [27]. To adapt to the specific environment on board the questionnaire was slightly altered for the maritime setting. Frequency and duration of usage (were differentiated into the three voyage episodes: port stay, sea passage, and at home. Reasons for **not** downloading an app and stopping the use were replenished by two items (*offline availability & availability of preferred language*) that were identified as important to seafarers by a third-party study of the EU project e-healthy-ship (S1 File).

After evaluating the data, important results of the questionnaire "*characteristics of health app use*" were compared with the sample of the study by Krebs & Duncan (2015) [27] in which 1,604 participants completed the questionnaire and 934 had downloaded an app. The mean age of those participants was 40.1 years (Standard Deviation (SD) 15.7) (18–81 years); 49.6% of the participants were women. Regarding ethnicity, 25.4% were black, 7.1% Asian, 35.5% white, 27.9% Latino/Hispanic, and 2.9% other.

Well-being was identified by using the WHO Well-being Index [28], a short five-item questionnaire designed to measure well-being [29]. Each question can be given values from 0 to 5 [30]. Summing up the scores for the answers gives a cumulative score, with a low total score corresponding to low well-being [31].

At the beginning of the survey, participants were informed in writing that their data would be treated confidentially and anonymously and that their participation was voluntary. Prior to the study, the participants were familiarized verbally with these aspects and received detailed information about the survey procedure, the security mechanisms to ensure the anonymity of the data (especially towards the employers), as well as which data would be collected and how it would be handled. It was also explicitly stated that participants had the option of leaving certain questions unanswered or withdrawing their consent at any time. This ensured informed verbal consent, which was also approved in this form by the ethics committee. The seafarers’ data was then collected anonymously on board each ship and transmitted together to the Institute of Occupational and Maritime Medicine (ZfAM). The institute then processed the data, ensuring anonymity throughout the entire analysis phase. The seafarers had 21 days to complete the questionnaire. The Ethics Commission of the Hamburg Medical Chamber (Ethik Kommission der Hamburger Ärztekammer) approved the study and informed consent process and gave a positive ethics vote (PV7174). The authors declare no conflict of interest. The funders had no role in the design of the study, in the collection, analyses, or interpretation of data, in the writing of the manuscript or in the decision to publish the results.

### Study population

976 seafarers on 65 merchant ships completed the questionnaire (response rate 75.1%; no data are available for an analysis of non-responders). 2 seafarers (0.2%) were 19 or younger, 196 between 20 and 29 (21.6%), 302 between 30 and 39 (33.3%), 263 between 40 and 49 (30.0%), 119 between 50 and 59 (13.1%) and 26 reported being 60 years or older (2.9%). A total of 6 (0.6%) crew members were female. The multicultural study population consisted of 207 (27.2%) Europeans and 553 (72.8%) non-Europeans. Filipinos were the most represented among the surveyed seafarers with 502 (66.1%), followed by 60 Ukrainians (7.9%) and 54 Romanians (7.1%). 638 seafarers reported having children (68.8%). 210 of the seafarers were single (22.8%), 696 were married (75.5%) and 16 reported that they were divorced (1.7%). 584 of the seafarers (66.7%) were non-smokers, and 69 (7.9%) used to smoke but reported that they no longer do so. The participant who smoked, smoked an average of 12.2 cigarettes per day (SD 7.0), had an average of 13.8 years of seafaring experience (SD 9.1), and had been on their respective ships for 5.3 months (SD 3.12). 355 of the respondents held an officer’s rank (38.9%). 557 (61.1%) were ratings (defined as seafarers who are not officers) (Table 1).

**Table 1:** Demographic data distinguished by occupation*.

912 seafarers^a^	*Officers (n = 355)*	*Ratings (n = 557)*
**Ranks,** n	201 nautical officers, 154 technical officers	284 ratings on deck, 153 machine ratings
		79 galley staff,
		31 Cadettes
		10 others
**Age,** n (%)		
< 40 years	210 (60.3%)*	278 (51.7%)*
≥ 40 years	138 (39.7%)*	260 (48.3%)*
**Gender,** n (%)		
Male	352 (99,4%)*	554 (99.5%)*
Female	2 (0.6%)*	3 (0.5%)*
**Origin,** n (%)		
Europeans	187 (61.9%)*	19 (4.2%)*
Non-Europeans	115 (38.1%)*	432 (95.8%)*
**Married,** n (%)	266 (77.1%)*	402 (73.9%)*
**Children,** n (%)	227 (65.0%)*	386 (70.8%)*
**Smoking status,** n (%)		
Never smoked	208 (61.1%)*	361 (70.4%)*
Former smoker	94 (28.1%)*	119 (23.2%)*
Smoker	33 (9.8%)*	33 (6.4%)*
**Alcohol consumption,** n (%) (n = 726)		
More than 0.5l beer, 0.3l wine or 0,08l liqueur per day	11 (3.6%)*	24 (5.7%)*
**WHO Well-being Score**, median (IQR)^**^	20.0 (16.0; 21.0)	20.0 (19.0; 24.0)
**Frequency of health app use** (at least once/day), n (%)		
At sea	53 (29.3%)*	30 (20.5%)*
In port	46 (27.4%)*	22 (19.8%)*
At home	80 (41.2%)*	52 (30.4%)*
**Duration of health app use** (at least more than 10 min when using it), n (%)		
At sea	44 (24.7%)*	50 (34.2%)*
In port	21 (12.9%)*	26 (24.5%)*
At home	83 (43.3%)*	74 (43.3%)*

^**a**^64 seafarers did not answer the question about their rank and were not displayed here

*Differences in the categories’ absolute sum are due to respective missing data

** Interquartile range

### Statistics

The statistical analysis was conducted using the open-source program R (version 4.3.0) provided by the R Foundation for Statistical Computing, established by the R Core team. Due to missing responses in the questionnaires, the sum of the absolute frequencies often does not correspond to the total frequency of the differentiation categories. The percentages, however, are expressed on 100% of the respondents of the respective question. To assess the statistical significance between data sets, Pearson’s Chi-Squared test or Fisher’s exact test was employed. The assumption of independence among individuals was made, and all tests did not necessitate a normal distribution of the data, which was generally not observed. The statistical testing was performed with a significance level (α) of 0.05.

To calculate the crude odds ratio (OR) along with 95% confidence intervals, binary logistic regression was utilized. For the multivariate analysis, linear logistic regression was employed, adjusting for key demographic/occupational variables (age, rank, ethnicity). Bivariate correlation was assessed via the Pearson correlation coefficient (PCC).

Even after excluding the six female seafarers, the results remained statistically significant. Therefore, they were retained in the dataset.

## Results

### Health app usage

In this survey, 940 seafarers (97.8%) reported having a smartphone on board and 133 participants owned a tablet (14.0%). The family or friends at home of 927 seafarers (96.9%) had a mobile device. A large proportion of respondents said they would never pay anything for a health app (288; 64.1%).

478 seafarers (52.5%) had already downloaded at least one health app in the past (downloaders). Multivariate analysis demonstrated that downloading an app was more strongly associated with being an officer than a rating (adjusted Odds Ratio (aOR) 1.67; 95% CI (1.13–2.50)). Moreover, downloaders were less likely to belong to older crew members (≥ 40 yrs.) (aOR 0.50; 95% CI (0.37–0.68)). No significant associations in the adjusted odds ratios were identified among the other subpopulations (ethnicity, marital status, WHO Well-being Score) (Table 2).

**Table 2:** Multivariate analysis of download behavior.

	*Downloader (478)*	*Non-downloader (433)*	*Crude OR (95% CI)*	*Adjusted OR (aOR) (95% CI)*
**Rank, n (%)**				
Officers (vs. Ratings)	215 (47.8%)	131 (31.9%)	1.96 (1.48–2.59)	1.67 (1.13–2.50)
**Age,** n (%)				
≥ 40 years (vs. < 40)	172 (37.6%)	212 (52.7%)	0.54 (0.41–0.71)	0.50 (0.37–0.68)
**Origin,** n (%)				
Europeans (vs. non- Europeans)	133 (34.5%)	70 (20.5%)	2.04 (1.46–2.87)	1.41 (0.91–2.20)
**Family status,** n (%)				
Married (vs. single)	339 (74.2%)	317 (77.5%)	0.98 (0.73–1.32)	1.21 (0.84–1.75)
**WHO Well-being Score**				
Well-being ≤ 13 (vs. well-being >13)	62 (13.4%)	33 (7.9%)	1.80 (1.16–2.84)	1.48 (0.87–2.56)

*adjusted for age, rank an ethnicity (if applicable)

### Download reasons and occupational differences

**Fig 1:**
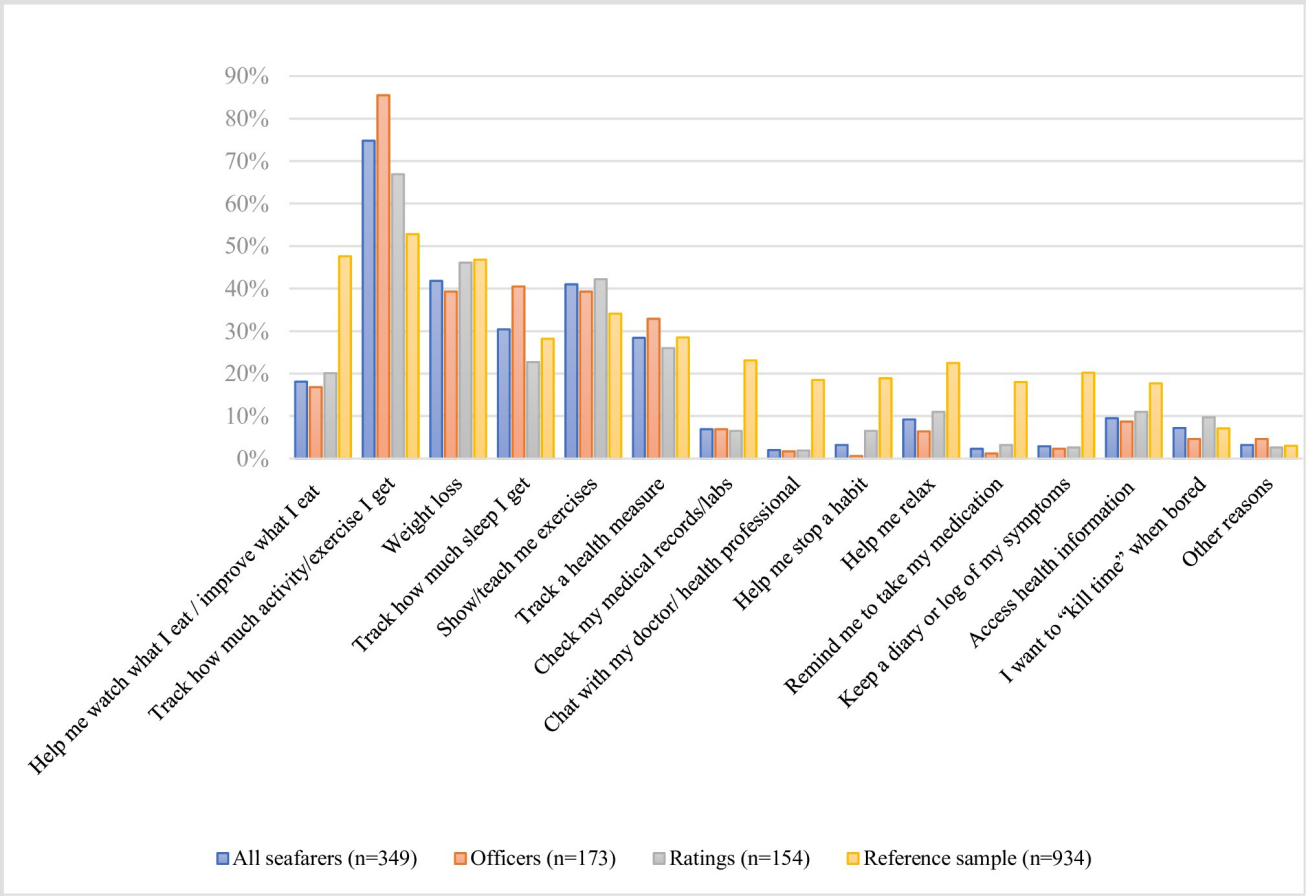
Reasons for downloading a health app. The chart shows the distribution of reasons for downloading a health app among the seafarers (divided by occupational group) and the reference sample by Duncan and Krebs (2015) [27], measured as a percentage of total respondents in each group. The data is categorized into four groups: all seafarers (n = 349*), officers (n = 173), ratings (n = 154), and the reference sample (n = 934). *22 respondents did not state their occupational rank and are included in the sample "all seafarers".

The top five reasons for downloading a health app among downloaders were *activity tracking* (261, 74.8%), *weight loss* (146, 41.8%), *getting taught/shown exercises* by the app (143, 41.0%), *sleep tracking* (106, 30.4%) and *tracking a health measure* (99, 28.4%) (Fig 1).

The proportion of officers who stated the reasons *activity tracking* (p< 0.001) and *sleep tracking* (p = 0.001) was significantly greater compared to the ratings. The proportion of ratings who stated that they downloaded a health app for *helping them to stop a habit* was significantly higher compared to the officers (p = 0.004).

Multivariate analysis demonstrated that being an officer (in contrast to ratings) was more strongly associated with being European (aOR 38.0; 95% CI (23.10–65.70)), and negatively associated with being 40 years or older (aOR 0.63; 95% CI (0.42–0.93)). Concerning the family status, WHO Well-being Score, frequency and duration of use within the three different voyage episodes (at sea, in port, and at home), no significant associations with the rank were found in multivariate analysis.

### Reasons for not downloading a health app

**Fig 2:**
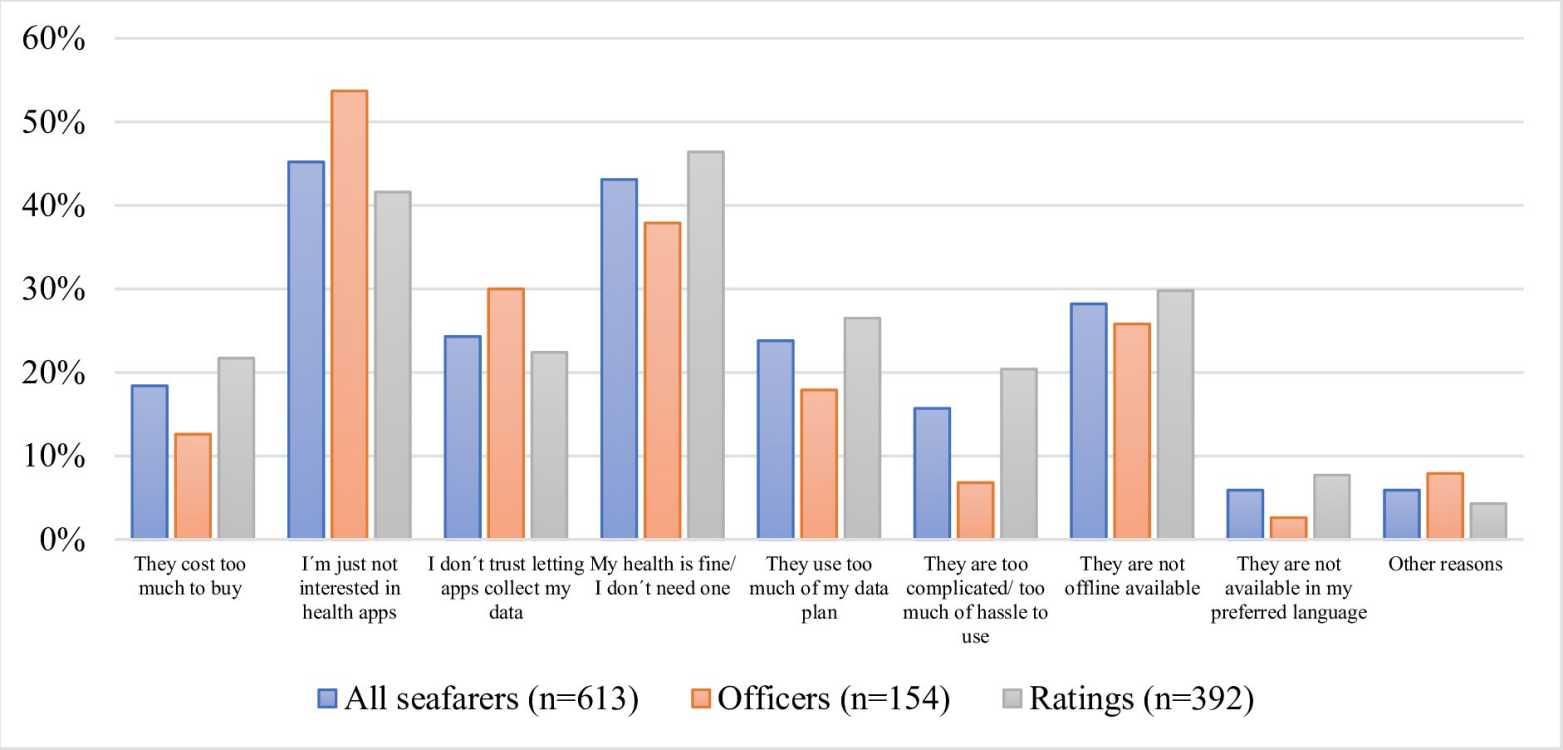
Reasons for not downloading a health app. This bar chart illustrates the percentage of respondents from different occupational groups: all seafarers (n = 613*), officers (n = 154), and ratings (n = 392). The chart highlights the distribution of reasons among these groups, measured as a percentage of total respondents in each group. *67 respondents did not state their occupational rank and are included in the sample "all seafarers".

The top five reasons for not downloading a health app were “*I am not interested in health apps*” (277, 45.2%), “*My health is fine/I don´t need one*” (264, 43.1%), “*They are not offline available*” (173, 28.2%), “*I don´t trust letting apps collect my data*” (149, 24.3%) and “*They use too much of my data plan*” (146, 23.8%) (Fig 2).

Officers stated significantly more often proportionally that they didn´t use certain health apps because they were not interested in them (p = 0.008). Ratings did not download certain health apps significantly more often proportionally due to the *costs* (p = 0.009) because the apps used *too much of their data plan* (p = 0.022), were *too complicated to use* (p< 0.001), or because they were *not available in their preferred languages* (p = 0.016).

### Reasons for stopping the use of a health app

51.1% (219) of downloaders, that answered this respective question, said they stopped the usage of at least one health app that they downloaded. No significant difference between officers and ratings could be identified in terms of the stop-use behavior (p = 0.922).

**Fig 3:**
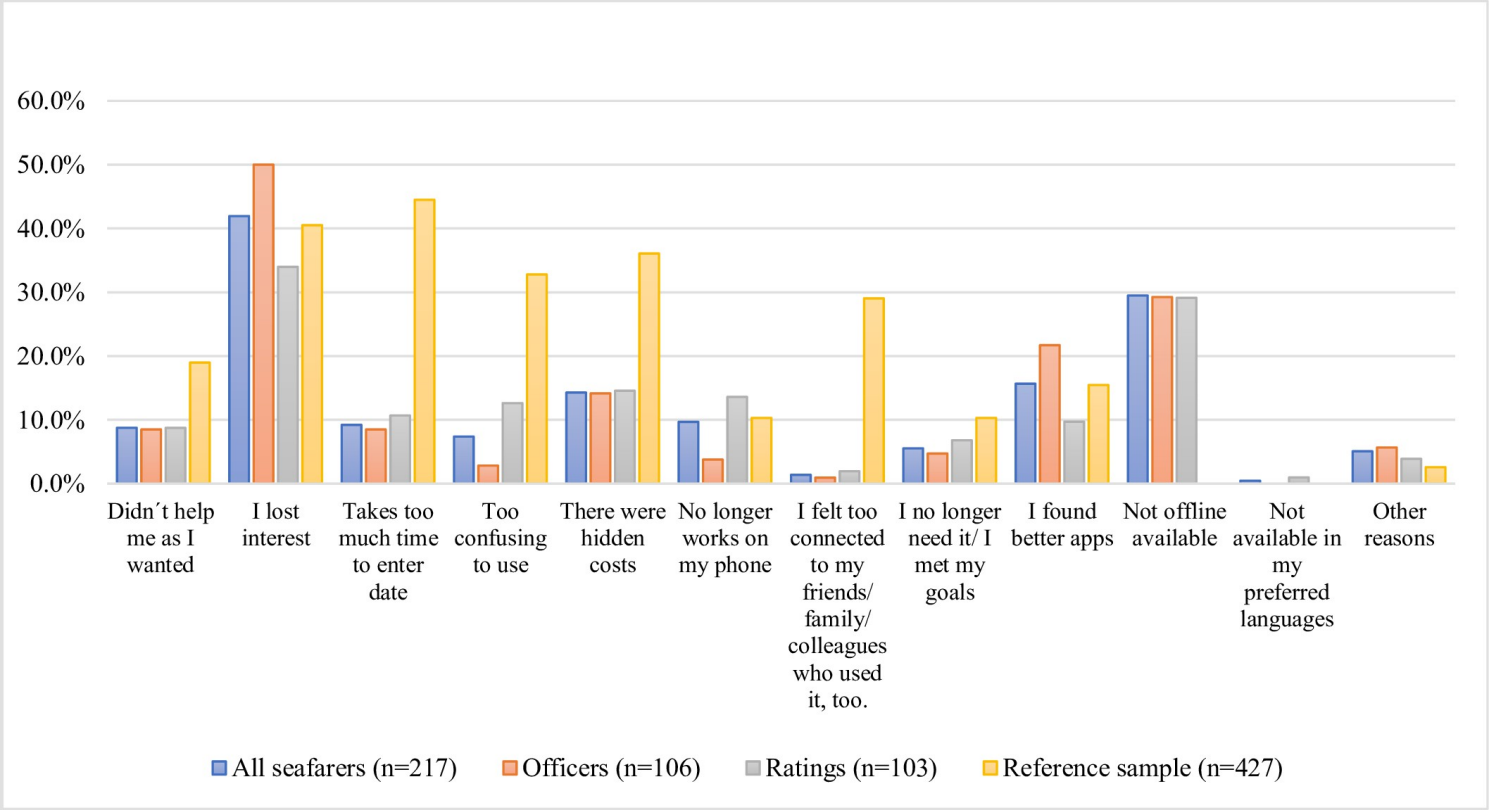
Reasons for stopping the use of a health app. This bar chart shows the percentage of respondents from various occupational groups who stopped using a health application divided by specific reasons. The data is categorized into four groups: all seafarers (n = 217*), officers (n = 106), ratings (n = 103), and the reference sample by Duncan and Krebs (2015) [27] (n = 427). Percentages are measured based on the total number of respondents in each group. *8 respondents did not state their occupational rank and are included in the sample "all seafarers".

The three most common reasons for stopping the use of an already downloaded app were the *loss of interest* (91, 41.9%), that the app was *not offline available* (64, 29.5%), and that the seafarers *found better apps* (34, 15.7%) (Fig 3). A significantly higher proportion of officers (compared to the ratings) stated a *loss of interest* (p = 0.025) and the *finding of better apps* (p = 0.022) as reasons. On the other hand, a higher proportion of the ratings stated that they stopped the usage because the apps were *too confusing to use* (p = 0.009) or that they *did not work any longer on their phones* (p = 0.013).

### Comparison with the reference sample of the Krebs & Duncan study (2015) [27]

Compared to the reference sample, a significantly lower proportion of seafarers ever downloaded a health app (58.2% (934)) [27] vs. 52.5% (478)) (p-value< 0.001). Seafarers downloaded health apps to a higher proportion than the reference sample for activity tracking (74.8% vs. 52.8%; p<0.001) and teaching them exercises (41.0% vs. 34.1%; p = 0.022). Beyond that, apps serving the following purposes were all significantly more strongly represented among the land-based sample (all p<0.001): checking medical records/labs, chatting with a doctor/health professional, stopping a habit or relaxing, medication reminder, diary log of symptoms and source of health information were all significantly more strongly represented among the land-based sample (all p<0.001).

The Chi-Squared test found no significant difference in respect of no longer using an app once it had been downloaded between the seafarers (219; 51.1%) and the reference group (427; 45.7%) (p = 0.070).

### Frequency and duration of use

The frequency of use among the seafarers was highest at home (mean 2.81 ±1.39 (scale from 1–5; 1 = less than once a month; 2 = a few times a month; 3 = a few times each week; 4 = about one time each day; 5 = two or more times a day), followed by the frequency of use at sea (mean 2.49 ±1.31) and then in port (mean 2.35 ±1.35), but was significantly lower in all voyage episodes than the frequency of use in the reference collective (mean 3.79) (all p-values< 0.001). 87 of the respondents (25.8%) who had already downloaded a health app and answered this question said that they opened their health apps at least once per day at sea. 71 (24.2%) did so while in port. 138 (35.8%) opened health apps at least once a day while at home, where a significant difference could be identified between officers and the ratings, whereby the officers had a significantly higher ratio (p = 0.038).

The duration of use of health apps was greatest among the seafarers at home (mean 1.68 ±0.84 (scale from 1–3; 1 = 1–10 minutes per usage; 2 = 11–30 minutes per usage; 3 = more than 30 minutes per usage)), followed by the duration at sea (mean 1.36 ±0.63), and then the duration of use in port (mean 1.21 ±0.67)). Compared to the reference sample (mean 1.67), the duration of use of the seafarers differed significantly only at sea and in port (both p< 0.001).

29.1% of those who downloaded a health app and answered this question said they used their apps for more than 10 minutes at sea when using them, 17.0% did so in port and 43.6% did so at home. A significant difference could be identified between officers and ratings in terms of the duration of use in port and at home, whereas a significantly greater proportion of ratings used their apps for more than 10 min in port (p = 0.021) and a significantly greater proportion of officers used them more than 10 min at home (p< 0.001).

### WHO five well-being index

No bivariate correlation could be identified between the WHO Well-being Score and the frequency and duration of use of health apps in all three different voyage episodes (in port, at sea, and at home) (all p> 0.05 (PCC)).

Multivariate analysis also found no significant association between a WHO Well-being Score ≤ 13 and the frequency (at least once per day) and duration (minimum of 10 min. per usage) of use of the seafarers. Regarding the download behavior bivariate correlation (p = 0.009; correlation: 0.09) and univariate association (aOR 1.80; 95% CI (1.16–2.48)) produced significant results. In both instances being a downloader was more strongly associated/correlated with a WHO Well-being Score≤ 13. After adjusting for age, rank and ethnicity, however, no significant association could be identified anymore in the multivariate analysis (aOR 1.47; 95% CI (0.87–2.55)).

## Discussion

This study examined the usage of app-based health prevention in a maritime-specific environment. For this purpose, the possibilities, the usage behavior of health apps and the respective needs among seafarers were evaluated.

### Technical requirements

97.8% of the respondents stated to own a smartphone and 14.0% a tablet, which is the basic requirement for conducting app-based health prevention. This is well above the smartphone penetration in a previous population-based cross-sectional survey ashore, where a proportion of 82.6% [32] was estimated.

### Health app usage and download-behavior

In the scientific literature, no assessment of the use of health apps in seafarer collectives was found. Therefore, the unique results of this survey had to be compared with those of study samples living on land. Due to the maritime-specific composition of this seafarer sample (e.g. very high proportion of men, different age, and broad ethnic distribution), however, the comparability might be limited.

52.5% of the seafarers stated that they had already downloaded at least one health app in the past. Even though this was a smaller proportion than in the survey by Krebs & Duncan (2015) [27] (58.2%), it was a significantly higher proportion in contrast to three other studies conducted in France (2022), Germany (2017) and Hongkong (2017) [32–34], in which the percentage of downloaders ranged from 20.5% to 46.7%. The authors of the French study by Paradis et al. (2022) [32] even suspected that their survey sample consisted of rather technology-savvy and health-conscious participants and might therefore have an elevated percentage of downloaders compared to the general population.

The high proportion of downloaders among seafarers is even more striking, as the results of Paradis et al. (2022) [32] found a positive correlation between downloading an app and being female. The seafarer sample, however, consisted of 99.4% men. Additionally, the findings of Ernsting et al. (2017) [33] indicate that there is a connection between the use of health apps and chronic diseases ‐ this would be in line with the mobile health (mhealth) study conducted in France [32], which found that after "well-being, prevention, and fitness", "treatment, medication, and follow-up" were the top reasons for downloading health apps. Among seafarers, however, one can assume a healthy-worker-effect due to pre-employment examination and subsequent regular medical fitness examinations every two years [35], which results in a lower proportion of chronic diseases. Despite these facts, seafarers had a higher proportion of downloaders in respect to 3 out of 4 depicted health app studies. This could indicate a promising premise for health interventions among seafarers. However, even though there seems to have existed a certain level of interest in the past due to the high download rate, the current frequency and duration of use as well as the reasoning behind stopping the use of a health app or not downloading one will put this assumption into perspective as discussed further below.

### Occupational and demographic differences in health app usage and download-behavior

Downloaders were significantly less prevalent among the older crew members (≥40 y.). This finding is coherent with the findings of other studies [27, 32, 33] and indicates that health apps have to be made even more attractive to older people, particularly since health issues become more significant as people age, for example by focusing on other topics such as chronic and orthopedic diseases.

Officers had higher odds to be downloaders and vice versa. A correlation between the use/download of health apps and a higher income and level of education could already be identified in earlier surveys [27, 32, 33]. Paradis et al. (2022) suggested that people who hold managerial, intellectual, or middle-level jobs ‐ as officers do ‐ tend to use health apps more often than others. According to the authors, this can be partly explained by the higher use of smartphones among people in this socio-professional category and their higher use of apps in general. This might also be the case among seafarers as differences in the health behavior and awareness were already described among officers and ratings [13]. Another important factor besides the education might be the easier access to onboard internet for private use (which also facilitates the use of health apps) mainly for officers and Europeans (in European waters due to cheaper tariffs) [21]. That also clearly indicates a considerable social gradient between the cultural and occupational groups aboard (which is consistent with the literature [36, 37]) and might explain the existing results. The easier access to onboard internet might also be the reason why officers stated significantly less frequently that the apps would use too much of their data plan as reason for not downloading an app.

Another reason for the download behavior difference might be cultural, as being an officer was associated with being European (aOR 38.0; 95% CI (23.10–65.70)). A study by Katzler et al. (2019) [7] concluded that the need for intensified health education among the Gilbertese (citizens of the South Pacific island country: Republic of Kiribati) of merchant ships was significantly higher than among European crew members. This could indicate that facilitating the use of health apps on board is a good option for shipping companies willing to improve health prevention especially for the ratings and non-Europeans on board.

### Areas of interest for downloading an app

The seafarers surveyed showed a rather strong interest in few health app categories, in contrast to the more diversified and evenly distributed area of interest in the sample by Krebs & Duncan (2015) [27] (Fig 1). Among the top reasons for downloading an app, a strong emphasis on topics related to physical activity could be found. This largely corresponds to the results of other land-based studies on health app use [27, 32, 34], although the present findings showed that seafarers had a more elevated interest in those health topics compared to the land-based reference sample (*activity tracking* (74.8% vs. 52.8%; p<0.001) and *learning exercises* (41.0% vs. 34.1%; p = 0.022). This could be either an indication that seafarers are not aware of other health topics addressed by health apps, which we will discuss later or that for some seafarers the need for physical activity is not yet satisfied by the work and living environment on board. A study even concluded that seafarers do less sport on board than at home [17]. In view of this result, the shipping companies should ensure that the fitness rooms on board are well equipped to meet the crews’ needs. This appears to be a crucial health intervention as well-equipped fitness rooms could be identified as one of the most important motivators for regular exercise among seafarers [17, 38] and might motivate the use of sports-based health apps.

### Occupational differences in areas of interest and neglected areas of interest

*Sleep tracking* was also among the top five reasons for downloading a health app, especially among officers. This is consistent with the lack of sleep and risk of fatigue typical of seafarers [6, 39, 40]. For instance, sleep deprivation was reported by almost 56% of seafarers, and correspondingly to our findings, especially by deck officers [4].

Activity tracking was ticked more frequently by a larger proportion of officers, too, which may be related to the finding that officers, in contrast to ratings, use sports to compensate for stress more regularly [38]. *Helping to stop a habit* was significantly more frequently downloaded among ratings. This might be due to the fact that smoking seems to be significantly more prevalent among non-officers [13]. However, only 6.5% of the ratings stated this download reason (compared to only 0.6% of the officers) which is a rather small percentage compared to 44% of smokers identified among seafarers by Hjarnoe & Leppin (2014) [13].

In general, it is striking that many fields of interest seem to almost be neglected or underrepresented by the seafaring sample when compared to the reference population as demonstrated in this study, like apps for *checking medical records/labs*, *helping to stop a habit* and *accessing health information*. Especially prominent is that the reason "*help me relax*" (9.2%) was also rarely ticked, even though it is well documented in literature that coping with stress is a challenge in a seafarer’s working routine [4, 41]. It is not clear yet whether seafarers simply do not want to use those types of health apps, whether they are not aware that health apps addressing those topics also exist, whether they possess a rather low health literacy (meaning they are not sufficiently informed, for instance, about the harms smoking and stress can cause) or whether there is simply no need. The last reason might be true for apps for *checking medical records/labs* or *medication intake* as the examined sample benefits from a healthy worker effect as already established above. Concerning the topics of relaxation and smoking cessation, it cannot be dismissed as easily as “not needed” given the amount of evidence in the literature regarding smoking and stress issues among seafarers. Further research is necessary in this regard, particularly by incorporating the potential long-term effects of health apps into the expanding body of fatigue studies that assess anti-fatigue measures. For now, education, promotion of the exchange of app experiences, app recommendations by shipping companies and health professionals, and app overviews might help to direct the focus towards apps that address health issues that seafarers suffer from.

### Reasons for not downloading or stopping the use of health apps

More than 50% of downloaders have stopped the use of a health app, which is consistent with the reference sample of Krebs & Duncan (2015) [27] and current literature. The top reason for not downloading as well as stopping the use of an app was a loss of interest (when already downloaded) (41.9%) or absence of interest (when it comes to initially downloading an app) (45.2%), especially among the officers. This is a phenomenon that is observed among many users of health apps and is consistent with the previous literature [27, 33, 42, 43]. Vaghefi (2019) [42] identified two dimensions influencing the stop-use behavior: users’ experience (*users’ assessment of mhealth app and its capabilities*) and intent (*their persistence in their health goals*). The users´ assessment can be influenced by the health app itself. A selection or recommendation, offered by health professionals or the shipping company of health apps with high appeal might help counteract this problem. In order to improve the seafarers´ persistence in their health goals the shipping companies could offer incentives for regular health app use or promote buddy team programs for health apps as competing and comparing with other crew members and exchanging experiences might work as additional motivation [43]. Moreover, younger seafarers could be paired with older crew members to help them overcome technical hurdles.

Offline availability of an app was also stressed as an important reason to stop using a health app or not even downloading it regardless of the rank. This poses a maritime-specific problem that also emerged in the unpublished third-party-funded study e-healthy-ship. Since many health apps use mobile data, the use of health apps is often complicated and expensive at sea. A data volume quota linked to health apps and offered by the shipping companies might motivate more seafarers to use this kind of support and lead to more awareness of health issues in the crew. Additionally, greater transparency regarding offline usability on the part of providers could be beneficial.

### Occupational differences in reasons for not downloading or stopping the use of health apps

It could be observed that ratings stated a loss of interest (34.0%) for stopping the use and an absence of interest (41.6%) for downloading an app significantly less frequently than officers (50.0%; p = 0.025 and 53.7%; p = 0.008). However, despite the lower amount among the ratings it should still be kept in mind that the issue of interest poses a tremendous problem since those answers still ranked among the top two reasons. One of the reasons why those answers were not as prevalent among the ratings might be rather due to other issues that hinder non-officers from using health apps. For instance the ratings´ answers suggest that their use of health apps is also limited due to their socio-economic situation (*they cost too much to buy; they no longer work on my phone)*. Licenses for paid apps bought by the shipping companies and issued to the crews could at least fix the economic problems caused by paid apps. The reason that apps don´t work anymore on their phones could possibly be due to old operating systems no longer supporting modern health apps or low-quality health apps that don´t get updated and therefore don´t work anymore on newer phones. This needs to be further investigated as the solution depends on the cause. The latter problem could be tackled by education about and recommendations of high-quality apps that are regularly maintained by the providers.

Even though ratings stated significantly more often that language barrier was a factor for not downloading an app, it was among the less mentioned reasons after all and supposedly does not pose a major obstacle.

Another reason stated by the ratings more often addressed a problem related to the users’ experience established by Vaghefi and Tulu (2019) [42]: apps were said to be *too complicated* or *too confusing*. This could be explained–besides by the low user-friendliness of apps–by the higher demand for more passive recreation in ratings due to frequent exposure to straining, even in their free time on board [38]. The use of health apps could therefore impose a perceived increase in effort, with which they do not want to waste their already limited free time. To counteract this problem the shipping company should recommend apps that meet the needs, in this case specifically relaxation apps. Another possibility would be a lack in the proficiency of mobile phone usage. This however seems to be rather unlikely as results of a study by Arslan et al. (2023) [26] indicate that the mobile proficiency among the examined seafarers seem to be rather high, even surpassing the proficiency of a land-based reference sample.

“I felt too connected with my friends” was relatively rarely given as a reason for stopping the use. This might be due to the fact, that seafarers already have scarce contact with their relatives [3].

In conclusion, it is essential to address both major issues, such as the absence of interest when initially downloading an app and the offline availability, as well as to implement tailored future intervention strategies for each professional group due to their differing needs, specifically to the target group of crew members (simple language, affordable acquisition).

### Frequency and duration of use

Seafarers used their apps for the longest time and most frequently at home, followed by the duration and frequency at sea and then in port. This can probably be explained by the increased workload in the port [44] as this voyage episode was stated as most stressful by seafarers [41] ‐ especially by nautical officers, which might also explain that this is the only episode in which ratings use health apps longer than officers.

Whether the available free time is also one of the main reasons that limit the use on board or whether the lacking offline availability at sea predominates here could not be determined in this study. In total it should be highlighted that seafarers generally have a higher interest or time to use health apps at home and only to a less extent during their stay on board probably due to the specific work-related circumstances. However, in comparison with the reference collective by Krebs & Duncan (2015) [27], both duration and frequency (except for duration at home) were significantly lower which might suggest that the limiting factors and reasons for not downloading/stopping the use of a health app pose bigger barriers in a maritime specific environment than for a land-based population.

### WHO five well-being index

Interestingly no correlation between the WHO Well-being score and the frequency and duration of usage could be identified. Similarly, no association could be identified in the multivariate analysis between the download-behavior and the WHO Well-being Score ≤ 13. This contradicts the results of the systematic review by Han & Lee (2018) [22] that suggest that the use of mobile health applications has a positive impact on health-related behaviors and clinical health outcomes. The high prevalence of stopping the use of health apps and the reduced use frequency and duration among the seafarers at sea might impair the effectiveness, however.

Univariate analysis, however, could find a negative association, which indicates that seafarers who felt uncomfortable had downloaded health-apps more frequently. This could be explained by the possible higher need and interest of health apps among individuals with lower well-being. Whether the negative correlation can solely be attributed to the download behavior or to the other factors of the adjustment (such as the rank), could not be identified. Further research has to be done in this regard. Self-assessment of a limited well-being by seafarers should therefore ideally entail access to information about possible causes to recommend specific health-apps to counteract.

## Strengths & limitations

This study assessed for the first time the usage and needs for health apps in seafaring by employing a relatively large sample size. The participation rate on this study was high leading to highly reliable estimates of survey responses. In terms of limitations, the questionnaire is a self-report measure and included only seafarers on board of one German shipping company, which is a common limitation in the field of maritime research. Moreover, due to the collaboration between shipping companies and crewing agencies in various countries, there exists a significant international diversity in the composition of the crews’ nationalities." Thus, general statements about other seafaring samples are possibly limited. Additionally, there is a possibility of biases such as self-selection bias among participants or social desirability bias in self-reported health behaviors. Although self-selection could influence the results, the authors believe that this bias has little impact on the validity of the results based on the following observation: Seafarers often welcome opportunities for engagement, which is reflected in the high response rate, suggesting a broad interest across the population studied. In regards to the social desirability bias, the extensive educational efforts (flyers, informational letters, informative discussions) concerning the study and the data protection concept (anonymity and confidentiality ensuring that supervisors/the shipowner cannot attribute data to individuals) suggest that no such bias is likely to have occurred. Furthermore, this study bases on a cross-sectional design so that longitudinal or interventional effects cannot be determined. Moreover, the unique composition of the sample (e.g. multicultural, very low ratio of women, few seafarers ≥ 60 y.) limits the comparability with land-based collectives as performed here. However, particularly these specific characteristics of the collective provide a more accurate representation of the typically multicultural seafaring population onboard, enhancing the validity of the study. In spite of the limitations in generalizability, the findings offer valuable insights into the specific behaviors and needs of seafarers concerning health apps, which can inform tailored interventions and policy-making in the maritime industry.

## Conclusion

The preconditions, such as owning a mobile device and proficiency in using mobile technology [26], seem to be in place as well as a basic awareness of and ‐ at least initial ‐ interest in health apps, considering amount of health app downloaders. Thus, the findings of this study suggest that health apps might serve as a useful tool for health promotion among seafarers. However, these existing and promising requirements are encountering obstacles that appear to prevent the success of an app-based preventive healthcare to date and result in below-average usage. This might also be one of the reasons why no clear correlation was found between the frequency/duration of use and the well-being. One of the major obstacles identified in this study is the lack or loss of interest in health apps, which is also a well-described phenomenon in the literature. However, as seafarers use health apps even less frequently while onboard than at home, maritime and occupational-specific limitations such as having limited spare time and being offline for long periods of time appear to have a significant negative impact, too. Economical and educational issues (e.g. paid apps, outdated phones, language skills) come into play as well, especially for ratings. A strong focus on apps related physical activity also indicates a lack of awareness concerning other health topics addressed by health apps.

To overcome these barriers and make an app-based health prevention more attractive to seafarers, shipping companies could offer internet contingents/allowances that give seafarers more constant access to apps. General education about the benefit and available health topics of health apps, guidance on selecting high-quality health apps, or even a choice of preselected apps might be helpful, too. This approach might make seafarers more persistent in using health apps and therefore increase the chance of health benefits arising from them. Providing licenses of apps purchased by the shipping companies and issued to the seafarers could help address the economic issue highlighted in the ratings. A recommendation of health apps, selected by health professionals, given to seafarers with a strong focus on quality and appeal, might help to overcome the issue of lack or loss of interest. Although the implementation of an app-based health promotion faces many obstacles, it seems to offer a promising approach to offer seafarers continuous, personal health prevention.

In addition, regulatory bodies, like the International Maritime Organization (IMO), might consider officially recommending the integration of health apps into maritime health management practices. This endorsement would of course be dependent on evidence-based findings from future studies proving their efficacy and positive health impacts. This highlights the relevance of ongoing research in this area. This would not only improve the well-being of seafarers but also add to the development of health care policies within maritime sector.

## Supporting information

S1 File Shortened version of the questionnaire “characteristics of health app use” by Krebs & Duncan [27]. (PDF)

## References

[1] Oldenburg M, Jensen HJ, Latza U, Baur X. Seafaring stressors aboard merchant and passenger ships. Int J Public Health. 2009;54(2):96–105. doi: 10.1007/s00038-009-7067-z 19288290

[2] Carotenuto A, Molino I, Fasanaro AM, Amenta F. Psychological stress in seafarers: a review. Int Marit Health. 2012;63(4):188–94. 24595974

[3] Jensen HJ, Oldenburg M. Objective and subjective measures to assess stress among seafarers. Int Marit Health. 2021;72(1):49–54. doi: 10.5603/IMH.2021.0007 33829473

[4] Oldenburg M, Jensen HJ. Stress and strain among seafarers related to the occupational groups. Int J Environ Res Public Health. 2019;16(7):1153. doi: 10.3390/ijerph16071153 30935082 PMC6480598

[5] Dohrmann SB, Leppin A. Determinants of seafarers’ fatigue: a systematic review and quality assessment. Int Arch Occup Environ Health. 2017;90(1):13–37. doi: 10.1007/s00420-016-1174-y 27804037

[6] Jepsen JR, Zhao Z, van Leeuwen WM. Seafarer fatigue: a review of risk factors, consequences for seafarers’ health and safety and options for mitigation. Int Marit Health. 2015;66(2):106–17. doi: 10.5603/IMH.2015.0024 26119681

[7] von Katzler R., Zyriax B.C., Jagemann B. et al. Lifestyle behaviour and prevalence of cardiovascular risk factors ‐ a pilot study comparing Kiribati and European seafarers. BMC Public Health 19.2019;855. doi: 10.1186/s12889-019-7186-2 31262273 PMC6604182

[8] Hoyer JL, Hansen H. Overweight among Nordic male seafarers. 8th International Symposium of Maritime Health 2005, Rijeka, Croatia.

[9] Pancic M, Ricka-Zauhar Z, Blazevic M. Analysis of risk factors and assessment of exposure to coronary diseases in seamen. 8th International Symposium of Maritime Health 2005, Rijeka, Croatia

[10] Nittari G., Tomassoni D., Di Canio M. et al. Overweight among seafarers working on board merchant ships. BMC Public Health 19.2019;45 doi: 10.1186/s12889-018-6377-6 30626365 PMC6327391

[11] Oldenburg M, Jensen HJ, Latza U, Baur X. Coronary risks among seafarers aboard German-flagged ships. Int Arch Occup Environ Health. 2008;81(6):735–41. doi: 10.1007/s00420-007-0261-5 17909838

[12] Oldenburg M. Risk of cardiovascular diseases in seafarers. Int Marit Health. 2014;65(2):53–7. doi: 10.5603/IMH.2014.0012 25231325

[13] Hjarnoe L, Leppin A. A risky occupation? (Un)healthy lifestyle behaviors among Danish seafarers. Health Promot Int. 2014;29(4):720–9. doi: 10.1093/heapro/dat024 23630132

[14] Pougnet R, Pougnet L, Loddé B, Canals L, Bell S, Lucas D, et al. Consumption of addictive substances in mariners. Int Marit Health. 2014;65(4):199–204. doi: 10.5603/IMH.2014.0038 25522703

[15] Hjarnoe L, Leppin A. What does it take to get a healthy diet at sea? A maritime study of the challenges of promoting a healthy lifestyle at the workplace at sea. Int Marit Health. 2014;65(2):79–86. doi: 10.5603/IMH.2014.0018 25231331

[16] Babicz-Zielinska E, Zabrocki R. Assessment of nutrition of seamen and fishermen. Rocz Panstw Zakl Hig.1998;49:499–505. 10224895

[17] Geving IH, Jørgensen KU, Thi MS, Sandsund M. Physical activity levels among offshore fleet seafarers. Int Marit Health. 2007;58(1–4):103–14. 18350980

[18] Carter T, Karlshoej K. The design of health promotion strategies for seafarers. Int Marit Health. 2017;68(2):102–107. doi: 10.5603/IMH.2017.0019 28660613

[19] Carter T. Mapping the knowledge base for maritime health: 3 illness and injury in seafarers. Int Marit Health. 2011;62(4):224–40. 22544497

[20] Baygi F, Djalalinia S, Qorbani M, Dejman M, Nielsen JB. Lifestyle interventions in the maritime settings: a systematic review. Environ Health Prev Med. 2020;25(1):10. doi: 10.1186/s12199-020-00848-7 32234023 PMC7110816

[21] Oldenburg M, Jensen HJ. Needs and possibilities for ship’s crews at high seas to communicate with their home. Int J Occup Med Environ Health. 2019;32(6):805–15. doi: 10.13075/ijomeh.1896.01436 31663520

[22] Han M, Lee E. Effectiveness of mobile health application use to improve health behavior changes: a systematic review of randomized controlled trials. Healthc Inform Res. 2018;24(3):207–26. doi: 10.4258/hir.2018.24.3.207 30109154 PMC6085201

[23] Battineni G, Di Canio M, Chintalapudi N, Amenta F, Nittari G. Development of physical training smartphone application to maintain fitness levels in seafarers. Int Marit Health. 2019;70(3):180–186. doi: 10.5603/IMH.2019.0028 31617935

[24] Stannard S, Vaughan C, Swift O, Robinson G, Altaf SA, McGarry A. Women seafarers’ health and welfare survey. Int Marit Health. 2015;66(3):123–38. doi: 10.5603/IMH.2015.0027 26394312

[25] Heydari E, Dehdari T, Solhi M. Can adopting skin cancer preventive behaviors among seafarers be increased via a theory-based mobile phone-based text message intervention? A randomized clinical trial. BMC Public Health. 2021;21(1):134. doi: 10.1186/s12889-020-09893-x 33446158 PMC7807693

[26] Arslan LC, Dengler D, Belz L, Neumann FA, Zyriax BC, Harth V, et al. Exploration of Seafarers’ Mobile Proficiency as a Prerequisite for Possible Health App-based Health Promotion on Board. Inquiry. 2023 Jan-Dec;60:469580231206264. doi: 10.1177/00469580231206264 37909669 PMC10621288

[27] Krebs P, Duncan DT. Health app use among US mobile phone owners: a national survey. JMIR Mhealth Uhealth. 2015;3(4):e101. doi: 10.2196/mhealth.4924 26537656 PMC4704953

[28] Brähler E, Zenger M, Kemper CJ. Psychologische und sozialwissenschaftliche Kurzskalen: Standardisierte Erhebungsinstrumente für Wissenschaft und Praxis (Psychological and social science short scales: standardized survey instruments for science and practice). MWV.2015;ISBN 978-3-95466-194-7:344.

[29] Nixdorff U. Check-Up-Medizin: Prävention von Krankheiten–Evidenzbasierte Empfehlungen für die Praxis (Disease prevention–evidence-based recommendations for practice). Georg Thieme Verlag. 2009;ISBN 978-3-13-170041-4:82.

[30] Bech P. Measuring the dimensions of psychological general well-being by the WHO-5. QoL Newsletter. 2004;32:15–6.

[31] Köllner V, Schauenburg H. Psychotherapie im Dialog–Diagnostik und Evaluation (Psychotherapy in dialogue ‐ diagnostics and evaluation). Georg Thieme Verlag. 2012;ISBN 978-3-13-170041-4:82.

[32] Paradis S, Roussel J, Bosson JL, Kern JB. use of smartphone health apps among patients aged 18 to 69 years in primary care: population-based cross-sectional survey. JMIR Form Res. 2022;6(6):e34882. doi: 10.2196/34882 35708744 PMC9247815

[33] Ernsting C, Dombrowski SU, Oedekoven M, O Sullivan JL, Kanzler M, Kuhlmey A, et al. Using smartphones and health apps to change and manage health behaviors: a population-based survey. J Med Internet Res. 2017;19(4):e101. doi: 10.2196/jmir.6838 28381394 PMC5399221

[34] Shen C, Wang MP, Chu JT, Wan A, Viswanath K, Chan SSC, et al. Health app possession among smartphone or tablet owners in Hong Kong: Population-based survey. JMIR Mhealth Uhealth. 2017;5(6):e77. doi: 10.2196/mhealth.7628 28583905 PMC5476868

[35] Oldenburg M, Jensen HJ. Are there differences between officers and ratings on merchant vessels concerning effort-reward imbalance: a cross-sectional maritime field study. Int Arch Occup Environ Health. 2022;95(1):131–40. doi: 10.1007/s00420-021-01779-8 34714395 PMC8755692

[36] Slišković A, Penezić Z. Testing the associations between different aspects of seafarers’ employment contract and on-board internet access and their job and life satisfaction and health. Arh Hig Rada Toksikol. 2016;67(4):351–363. doi: 10.1515/aiht-2016-67-2785 28033098

[37] Harris R. Communications for seafarers ‐ More needs to be done. International Seafarers’ Welfare and Assistance Net-work (ISWAN); 2019 [cited 2023 Jun] Available from: https://www.seafarerswelfare.org/news/2013/communications-for-seafarers-more-needs-to-be-done

[38] Oldenburg M., Jensen HJ. Recreational possibilities for seafarers during shipboard leisure time. Int Arch Occup Environ Health 92. 2019;1033–9 doi: 10.1007/s00420-019-01442-3 31114964

[39] Allen P, Wadsworth E, Smith A. The prevention and management of seafarers’ fatigue: a review. Int Marit Health. 2007;58(1–4):167–77. 18350986

[40] Wadsworth EJ, Allen PH, McNamara RL, Smith AP. Fatigue and health in a seafaring population. Occup Med (Lond). 2008;58(3):198–204. doi: 10.1093/occmed/kqn008 18310605

[41] Oldenburg M, Jensen HJ. Stress and strain among merchant seafarers differ across the three voyage episodes of port stay, river passage and sea passage. PLoS One. 2019 Jun 4;14(6):e0217904. 31163071 10.1371/journal.pone.0217904 PMC6548393

[42] Vaghefi I, Tulu B. The continued use of mobile health apps: insights from a longitudinal study. JMIR Mhealth Uhealth. 2019;7(8):e12983. doi: 10.2196/12983 31469081 PMC6740166

[43] Perez S. Nearly 1 in 4 people abandon mobile apps after only one use. Join TechCrunch+. 2019 [cited 2023 Jun] Available from: https://techcrunch.com/2016/05/31/nearly-1-in-4-people-abandon-mobile-apps-after-only-one-use/?guccounter=1

[44] Cezar-Vaz MR, Bonow CA, Almeida MC, Sant’Anna CF, Cardoso LS. Workload and associated factors: a study in maritime port in Brazil. Rev Lat Am Enfermagem. 2016 Nov 28;24:e2837. doi: 10.1590/1518-8345.1347.2837 27901222 PMC5172618

