# Supplementary material for: Possibilities, usage and needs of an app-based health prevention among seafarers
Source: PLoS One. 2024 Nov 27;19(11):e0310440. doi: 10.1371/journal.pone.0310440 (PMC11602079; doi:10.1371/journal.pone.0310440)
Supplement: S1 File — (PDF) [file pone.0310440.s001.pdf]

Supporting Information 1. Shortened version of the questionnaire “*characteristics of health app use*” by Krebs & Duncan [27].

Item:	No	Yes
1. Do you have a smart phone on board?	☐	☐
2. Do you have a tablet on board?	☐	☐
3. Do your friends/family members at home have a smartphone, tablet or other IT-device when you are on board?	☐	☐

4. Have you ever downloaded an ´app´ to track anything related to your health?	☐ No
	☐ Yes
5 . Please check off all the reasons you have used health apps for.	☐ Help me watch what I eat / improve what I eat
	☐ Track how much activity / exercise I get
	☐ Weight loss
	☐ Track how much sleep I get
	☐ Show / teach me exercises
	☐ Track a health measure (such as blood pressure, blood sugar)
	☐ Check my medical records / labs
	☐ Chat with my doctor/s or another health professional
	☐ Help me stop a habit (such as smoking)
	☐ Help me relax (like a meditation or yoga app)
	☐ Remind me to take my medication (such as blood pressure or HIV/AIDS medication)
	☐ Keep a diary or log of my symptoms
	☐ Access health information on symptoms, treatments, diagnoses, etc.
	☐ I want to “kill time” when bored.
	☐ Other
6. Please check off the reasons why you have not downloaded a health app.	☐ They cost too much to buy
	☐ I’m just not interested in health apps
	☐ I don’t trust letting apps collect my data
	☐ My health is fine / I don’t need one
	☐ They would use too much of my data plan
	☐ They are too complicated/ too much of a hassle to use

<table>
<tr><td rowspan="3"></td><td colspan="4">☐ They are not offline available</td></tr>
<tr><td colspan="4">☐ Not available in my preferred language(s)</td></tr>
<tr><td colspan="4">☐ Other</td></tr>
<tr><td rowspan="8">7. What would be the maximum amount you would pay for a health-related app?

(in US$)</td><td colspan="4">☐ I wouldn't pay anything</td></tr>
<tr><td colspan="4">☐ Less than $1</td></tr>
<tr><td colspan="4">☐ $1- $1.99</td></tr>
<tr><td colspan="4">☐ $2-$3.99</td></tr>
<tr><td colspan="4">☐ $4-$5.99</td></tr>
<tr><td colspan="4">☐ $6-$9.99</td></tr>
<tr><td colspan="4">☐ $10-$19.99</td></tr>
<tr><td colspan="4">☐ $20+</td></tr>
<tr><td rowspan="6">8. On average, how often do you open or log on to use the health app you use most often…</td><td></td><td>… on board at sea?</td><td>… when being in port?</td><td>… at home?</td></tr>
<tr><td>Less than once a month</td><td>☐</td><td>☐</td><td>☐</td></tr>
<tr><td>A few times a month</td><td>☐</td><td>☐</td><td>☐</td></tr>
<tr><td>A few times each week</td><td>☐</td><td>☐</td><td>☐</td></tr>
<tr><td>About 1 time each day</td><td>☐</td><td>☐</td><td>☐</td></tr>
<tr><td>2 or more times a day</td><td>☐</td><td>☐</td><td>☐</td></tr>
<tr><td rowspan="4">9. On average, how many minutes do you spend using health-related smartphone apps on days that you use them…</td><td></td><td>… on board at sea?</td><td>… when being in port?</td><td>…at home?</td></tr>
<tr><td>1-10 minutes</td><td>☐</td><td>☐</td><td>☐</td></tr>
<tr><td>11-30 minutes</td><td>☐</td><td>☐</td><td>☐</td></tr>
<tr><td>more than 30 minutes</td><td>☐</td><td>☐</td><td>☐</td></tr>
<tr><td rowspan="2">10. Are there any health apps you downloaded and no longer use?</td><td colspan="4">☐ No, I still use all of them</td></tr>
<tr><td colspan="4">☐ Yes</td></tr>
<tr><td rowspan="6">11. For what reasons do you no longer use them?

(check off all that apply)</td><td colspan="4">☐ Didn’t help me as I wanted</td></tr>
<tr><td colspan="4">☐ Lost interest</td></tr>
<tr><td colspan="4">☐ Takes too much time to enter data</td></tr>
<tr><td colspan="4">☐ Too confusing to use</td></tr>
<tr><td colspan="4">☐ There were hidden costs</td></tr>
<tr><td colspan="4">☐ No longer works on my phone</td></tr>
</table>

	☐ I felt too connected to my friends / family / colleagues who used it, too
	☐ I no longer need it / I met my goals
	☐ Found better apps
	☐ Not offline available
	☐ Not available in my preferred language(s)
	☐ Other
